# Telomere length kinetics assay (TELKA) sorts the telomere length maintenance (*tlm*) mutants into functional groups

**DOI:** 10.1093/nar/gku267

**Published:** 2014-04-11

**Authors:** Linda Rubinstein, Lior Ungar, Yaniv Harari, Vera Babin, Shay Ben-Aroya, Gabor Merenyi, Lisette Marjavaara, Andrei Chabes, Martin Kupiec

**Affiliations:** 1 Department of Molecular Microbiology and Biotechnology, Tel Aviv University, Ramat Aviv 69978, Israel; 2 Faculty of Life Sciences Bar-Ilan University, Ramat-Gan, Israel; 3 Department of Medical Biochemistry and Biophysics and Laboratory for Molecular Infection Medicine Sweden (MIMS), Umeå University, Umeå 901 87, Sweden

## Abstract

Genome-wide systematic screens in yeast have uncovered a large gene network (the telomere length maintenance network or TLM), encompassing more than 400 genes, which acts coordinatively to maintain telomere length. Identifying the genes was an important first stage; the next challenge is to decipher their mechanism of action and to organize then into functional groups or pathways. Here we present a new telomere-length measuring program, TelQuant, and a novel assay, telomere length kinetics assay, and use them to organize *tlm* mutants into functional classes. Our results show that a mutant defective for the relatively unknown *MET7* gene has the same telomeric kinetics as mutants defective for the ribonucleotide reductase subunit Rnr1, in charge of the limiting step in dNTP synthesis, or for the Ku heterodimer, a well-established telomere complex. We confirm the epistatic relationship between the mutants and show that physical interactions exist between Rnr1 and Met7. We also show that Met7 and the Ku heterodimer affect dNTP formation, and play a role in non-homologous end joining. Thus, our telomere kinetics assay uncovers new functional groups, as well as complex genetic interactions between *tlm* mutants.

## INTRODUCTION

Telomeres are the specialized DNA–protein structures at the ends of eukaryotic chromosomes. Telomeres are essential for chromosomal stability and integrity, as they prevent chromosome ends from being recognized as double-strand breaks (DSBs) (1). In most eukaryotes the telomeric DNA consists of tracts of tandemly repeated sequences whose overall length is highly regulated (2). Telomeric DNA is synthesized by the enzyme telomerase, which copies a short template sequence within its own RNA moiety (3). In somatic cells, telomerase activity is repressed. Conventional DNA polymerases are unable to replicate the very ends of chromosomes due to their primer dependency; as a result, telomeres shorten with replicative age *in vitro* (4,5). Reactivation of telomerase in cultured cells results in extended life span leading to their apparent immortalization (6). Moreover, it has been shown that replenishing telomeres by an activated telomerase or by recombination (“Alternative Lengthening of Telomeres” or ALT) is one of the few essential steps that a normal human fibroblast cell must take on its way to become malignant (7). Thus, understanding how telomere length is monitored has significant medical implications especially in the fields of aging and cancer.

Much of what we know about the mechanisms that control telomere length homeostasis and telomere end protection comes from studies carried out in model organisms, such as fission and budding yeasts (8). Telomere size is maintained through a balance between activities that negatively and positively affect the activity of telomerase and of nucleases. The relative uniformity in telomere size is achieved by a mechanism able to ‘count’ telomere binding proteins (e.g. Rap1 in yeast and TRF1 in humans) that presumably affect chromatin structure and accessibility of the telomerase and nucleases to the chromosomal ends (9). However, our knowledge about the mechanisms that regulate telomere length is still very limited.

In yeast, telomerase is constitutively active and is required for the elongation of the shortest telomeres in the cell (10). Three large-scale systematic genetic screens uncovered a large network of genes (more than 400) that participates in controlling telomere length (11–13). These telomere length maintenance (*TLM*) genes have many different biochemical functions and localize to several compartments in the cell. Most of these genes were not previously known to play a role in telomere size homeostasis, and their mechanism of action is only now starting to be studied. The majority of the *TLM* genes identified in these screens are evolutionarily conserved and have human orthologs.

The genome-wide screens described have greatly increased the number of genes known to affect telomere length. The challenge ahead is to decipher their mechanism of action. An important step in that direction is to try to organize the mutants into coherent groups or pathways. Here we present a novel assay, TELKA (telomere length kinetics assay), and use it to organize *tlm* mutants into functional classes. Our results show that a mutant defective for the relatively unknown *MET7* gene has the same telomeric kinetics as *rnr1Δ yku70Δ* and *yku80Δ* mutants. The latter are defective for the Ku heterodimer, which plays a central role in telomere biology and in non-homologous end joining (NHEJ). Consistently, we show that Met7 plays a role in NHEJ and that Ku mutants are epistatic to *met7* with respect to telomere length maintenance. The *RNR1* gene encodes the large subunit of the ribonucleotide reductase (RNR) enzyme, the limiting step in dNTP synthesis (14). In its absence, telomeres are extremely short. Our results show a physical interaction between Met7 and Rnr1. Measurements of dNTP levels show that *met7* mutants have abnormal dNTP ratios; surprisingly, this dNTP phenotype can be suppressed by mutations in the Ku heterodimer. Thus, our telomere kinetics assay uncovers new functional groups, as well as complex genetic interactions between *tlm* mutants.

## MATERIALS AND METHODS

### Yeast strains

All strains used are derivatives of BY4741 (15). Standard yeast genetic procedures were used to create single and double mutants.

### Southern teloblots

Teloblots were carried out as previously described (13). Cells underwent nine consecutive re-streaks (∼25 generations each), and DNA was extracted from each passage. By re-streaking colonies of similar size, irrespectively of the time it took to reach that size, we ensured that all mutants underwent a similar number of cell divisions. A *Saccharomyces cerevisiae*-specific telomeric probe and a size marker were used as probes. The size-control probe is a specific region of chromosome II (positions 558490 to 559790) that detects two bands in the *Xho*I digested genomic DNA (2044 and 779bp long). Telomere length was measured with TelQuant, a VisualBasic6 program specifically developed for measuring telomere length in yeast (detailed in Supplementary Text1).

### TELKA

All the data measured by TelQuant analysis was analysed using Excel. Clusters were made using CLUSTER3 (16) (http://bonsai.hgc.jp/∼mdehoon/software/cluster/) and viewed by the JAVA TREEVIEW program (http://jtreeview.sourceforge.net/) (17). Preliminary analysis of mutants affecting known protein complexes showed that this method gave the best results. The 30 mutants analysed here were chosen based on the following criteria: (i) short mutants were analysed; (ii) one representative of each known complexes was chosen (with the exception of the mutants used for calibrating TELKA, which belonged to known complexes); (iii) their final telomere length was at least 10% shorter than the wt length. Comparing the intra-cluster Euclidian distances in the clusters obtained to a set of a million random permutations of the dataset resulted in an empirical *P*-value <10^−6^.

### dNTP level measurements

Measurements were as previously described (18).

### Western blots

Cells were collected by centrifugation, re-suspended in 600 μl of phosphate-buffered saline with 1% Triton X-100 (PBST), supplemented with a protease inhibitor cocktail (Roche) and subjected to mechanical rupture using glass beads. The cell debris was removed by centrifugation, and the supernatants were applied onto 0.1 M dithiothreitol, and incubated at 80°C for 10 min before sodium dodecyl sulphate-polyacrylamide gel electrophoresis (SDS-PAGE) (Resolving gel: 30% Acrylamide, 1.5 M Tris-HCl pH8.8, 10% SDS (pH 7.2), 9.7 ml H2O, 100 μl 10% APS and 10 μ TEMED. Stacking gel: 30% Bis/Acrylamide, 1 M Tris-HCl pH 6.8, 10% SDS (pH 7.2), 5.5 ml H2O, 800 μl 10% APS and 8 μl TEMED). The samples were run with SDS-PAGE buffer at 100 V until the samples have passed the stacking gel and then at 160 V until the samples have been fully separated. Transfer to nitrocellulose was done in transfer buffer (200 ml Methanol, 3.03 gr Tris Base, 14.4 gr Glycine) at 500 mAmp and verified by staining with Ponceau-S dye. The blot was blocked with Milk for at least 60 min at room temperature. Primary antibody was added for 12 h at 4°C. The blot was washed 3 × 5 min with TBST (Tris-Buffered Saline Tween-20) and secondary antibody was added for 1 h. The blot was washed 3 × 5 min with TBST and subjected to electro-chemi-luminiscence (ECL).

### Chromatin immunoprecipitation

Typically 50 ml of a log culture (5 × 10^7^ cells/ml) was cross-linked for 30 min in 1% formaldehyde. The cross-linker was quenched by the addition of Glycine to 125 mM and the cells were incubated for 5 min at room temperature. Cells were washed twice with TBS + 10% glycerol and vortexed for 45 min in 600 μl of lysis buffer (50 mM HEPES pH7.5, 1% triton, 0.1% SDS, 0.1% Deoxycholate, 2.5 mM EDTA, 0.5 M NaCl) supplemented with protease inhibitors (Roche) and glass beads. The crude lysate was sonicated to an average fragment size of 300 bp (15 × 10 s pulses at 80% power levels using a Sonic Vibra cell sonicator) after which the supernatant was clarified (14 000 rpm, 20 min). Protein concentrations were used to normalize all samples. Four hundred fifty microliter of the clarified lysates was used for immunoprecipitations. The immune complexes were retrieved using protein G beads (Adar Biotech) and washed using lysis buffer, wash buffer (250 mM LiCl, 0.5% NP40, 0.5% Deoxycholate, 5 mM EDTA) and TE. DNA was eluted, cross linking was reversed and the DNA was ethanol precipitated and resuspended in 50 μl of TE. One microliter was used for polymerase chain reaction (PCR). Every PCR reaction was carried out simultaneously on input DNA and on the relevant ImmunoPrecipitation to control for changes in PCR conditions. Reaction products were resolved on 2% agarose gel. Telomeric and non-telomeric (from the *SMC2* gene, located ∼60 kb from the telomere) PCR oligos were used. For more quantitative results, real-time PCR reactions were carried out using the following primers: Y′-element:5′-GGCTTGATTTGGCAAACGTT-3′, and 5′-GTGAACCGCTACCATCAGCAT-3′. ARO1:5′-GTCGTTACAAGGTGATGCC-3′, and 5′- CGAAATAGCGGCAACAAC-3′. The association of Ku70-Myc and Ku80-Myc with Y′-element telomeres was detected using Santa Cruz Mouse anti Myc monoclonal IgG antibodies (SC-40). The relative fold enrichment\depletion of the telomere-associated proteins Ku70 and Ku80 was calculated as follows: [tel-IP/ARO1-IP]/[tel-input/ARO1-input](19).

### Co-IP

One hundred milliliter of logarithmic cells was harvested, washed twice with water and re-suspended in 4 ml of phosphate buffered saline (PBS) buffer supplemented with 0.5% tween, 10% glycerol, 1 mM PMSF [Sigma] and protease inhibitors. An equal volume of glass beads (diameter 0.5 mm) was added. Breakage was achieved by vortexing for 60 min at 4°C. The supernatant was used for input and for immunoprecipitation. One microgram of antibody was added and incubated overnight at 4°C. Twenty microliter of protein A-Sepharose and G-Sepharose beads was added, and the incubation continued for 2 hr. The immunoprecipitates were washed five times for 5 min with PBS buffer and subsequently re-suspended in 40 μl SDS-PAGE sample buffer. 30–40 μl of the eluted proteins were analysed by SDS-PAGE and western blotting with anti Rnr1 (SC-11981 ENCO) antibody and anti-TAP (CAB1001 ThermoFisher) antibody.

### NHEJ assay

The assay was performed as described in (20). The plasmid used was pRS415 cut with restriction enzyme *Xho*I. In brief, the number of yeast transformants obtained with a linearized plasmid is compared to the number obtained with the uncut plasmid.

## RESULTS

### TelQuant

Accurate telomere length measurement in yeast is usually carried out by telomeric Southern blot analysis (teloblots) with a telomere-specific probe (13). The terminal restriction fragment appears in teloblots as a fuzzy smear, as it is composed of fragments derived from many telomeres in each cell, and from a large number of cells. Nevertheless, the size of telomeres in a given genetic background is remarkably stable, producing very repeatable results. In order to get an accurate reading of telomere length, we developed TelQuant, a graphical densitometry program programmed in VisualBasic6 language, able to acquire data in a semi-automated fashion (see Supplementary Text1).

**Figure 1.**
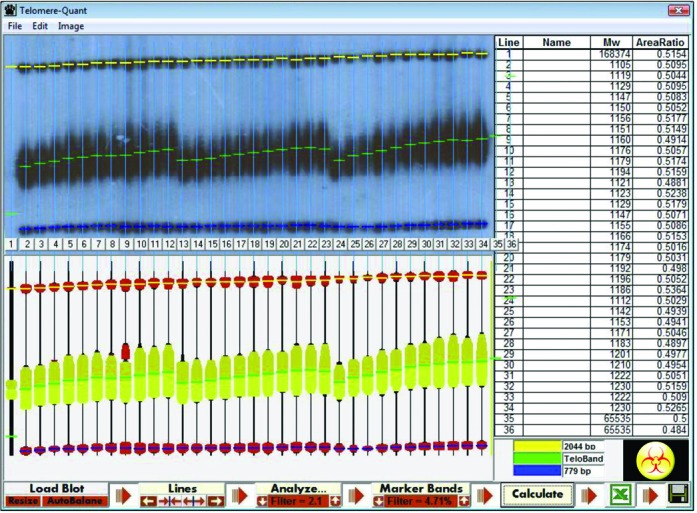
TelQuant. The TelQuant program scans teloblots and determines the median telomere length of each sample. The program automatically detects size markers and the terminal telomeric fragment, calculates telomere size and exports the results to an Excel sheet for additional processing.

We carry our teloblots by digesting the yeast DNA with *Xho*I and, after transfer, hybridizing the membrane with a probe composed of a telomere-specific region and a single fragment of chromosome II that generates, upon *Xho*I digestion, two bands of length 779 and 2044 bp, which serve as size markers. TelQuant scans the autoradiogram and calculates the mean and median telomere length of each lane by comparing their position relative to the two marker bands (Figure 1) (see Supplementary Text1).

In order to quantitate the accuracy and reproducibility of the results obtained with TelQuant, we took advantage of the fact that wild-type control lanes are present in each Southern blot carried out in the lab. Measurement of 103 different electrophoretic runs of the wt strain (BY7471) in 87 teloblots produced a telomere length of 352 ± 20 bp. Thus, telomere length measurements using TelQuant are highly reproducible and accurate, and they reveal that telomere length is tightly regulated in the wild-type strain. With a few exceptions (e.g. *rif1Δ* and *rif2Δ*), we found that *tlm* mutants, once they reach their final telomere length, are also very stable.

### TELKA arranges the *TLM* genes into functionally related groups

Telomere size is maintained through a balance between activities that negatively and positively affect telomere length: Telomerase activity adds telomeric repeats; concomitantly telomere sequences are lost (due to the end replication problem and to nuclease activity). These activities are controlled by a counting mechanism that is based on the number of telomeric repeats (21–23).

We assume telomere length in the wild-type strain to be in equilibrium. *tlm* mutations disturb this equilibrium, and therefore transiently shift the balance in one direction, causing telomere elongation or shortening. However, the homeostasis mechanisms continue to work, and the system regulates itself to a new, stable equilibrium point.

All the mutations uncovered in the genome-wide screen are recessive; thus, when crossing, for example, a mutant strain with short telomeres to a wild-type strain, the resulting heterozygous diploid, which starts with a mixture of short and normal-sized telomeres (inherited from the mutant and wt parent, respectively), returns to wt telomere length after a number of generations. Following meiosis, this heterozygous strain with telomeres of wt length produces gametes that are genetically either wt or mutant. Once the mutant cell starts to grow and divide, the telomeres shorten with each generation until they reach their new equilibrium point. Measuring telomere length from point zero (i.e. a spore) to the point at which a new equilibrium is achieved reveals a kinetic pattern for each mutant strain. We hypothesized that genes with similar roles in telomere length maintenance (e.g. members of the same complex or pathway) may demonstrate a similar length kinetic pattern. We thus measured the kinetics of telomere shortening in several *tlm* mutants freshly created after meiosis of heterozygous diploids. We call this methodology TELKA (telomere length kinetics assay).

To investigate whether our hypothesis regarding telomere length of members of the same complex is valid, we used TELKA to check if genes belonging to the same complex will present similar telomere kinetics. First, we chose several known complexes and checked the telomere kinetics of mutants affecting individual subunits. When analyzing 16 different mutants (representing six different protein complexes), TELKA was able to cluster them into six appropriate separate categories based on their telomere kinetics (Supplementary Figure S1A).

Encouraged by this result we chose additional *MATa* short *tlm* mutant strains (Supplementary Table S1), each carrying a gene deletion mutation marked by a KanMX marker which confers G418 resistance (24). These mutants were chosen among the short *tlm* mutants by applying two parameters: (i) they should show a significant (easily scored) telomere length shortening and (ii) whenever several mutants affecting a single protein complex were known, only one representative was chosen. The *tlm* mutants were mated with a *MATα* wild-type isogenic strain carrying a *ho::URA3* selectable marker and the *MFA1pr-HIS3* reporter (15). The *ho::URA3* allele has no phenotypic consequences, as all the strains used are heterothallic [and therefore carry a mutated HO gene, (ho-)]. Heterozygous diploids were selected on medium containing G418 and lacking uracil, and subjected to four consecutive re-streaks (∼100 generations) in order to ensure the restoration of wild-type length to all telomeres. The diploids were then subjected to meiosis, and single mutants were selected on synthetic medium lacking histidine and uracil and containing G418. This procedure selects for haploid derivatives carrying the *tlm* deletion. Individual colonies were then picked and streaked for ∼250 generations; telomere lengths were measured every ∼25 generations (i.e. every streak) by teloblots and quantified using the TelQuant software (Figure 1). Supplementary Figure S1B and C show two examples of *tlm* mutants that are deficient for the same protein complex: The *upf1(nam7), upf2(nmd2)* and *upf3* are part of the the nonsense-mediated decay pathway (25) and show similar kinetics. Similarly, the *mak10, mak3* and *mak31* proteins, all part of the N-terminal Acetyltransferase complex (26), show similar kinetics. We trained TELKA on the mutants affecting well-characterized complexes. Using kinetic information of the nine re-streaks gave a better separation into consistent groups than just using the final telomere length (e.g. *xrs2* gets separated from its partner *rad50* if only final length is considered).

**Figure 2.**
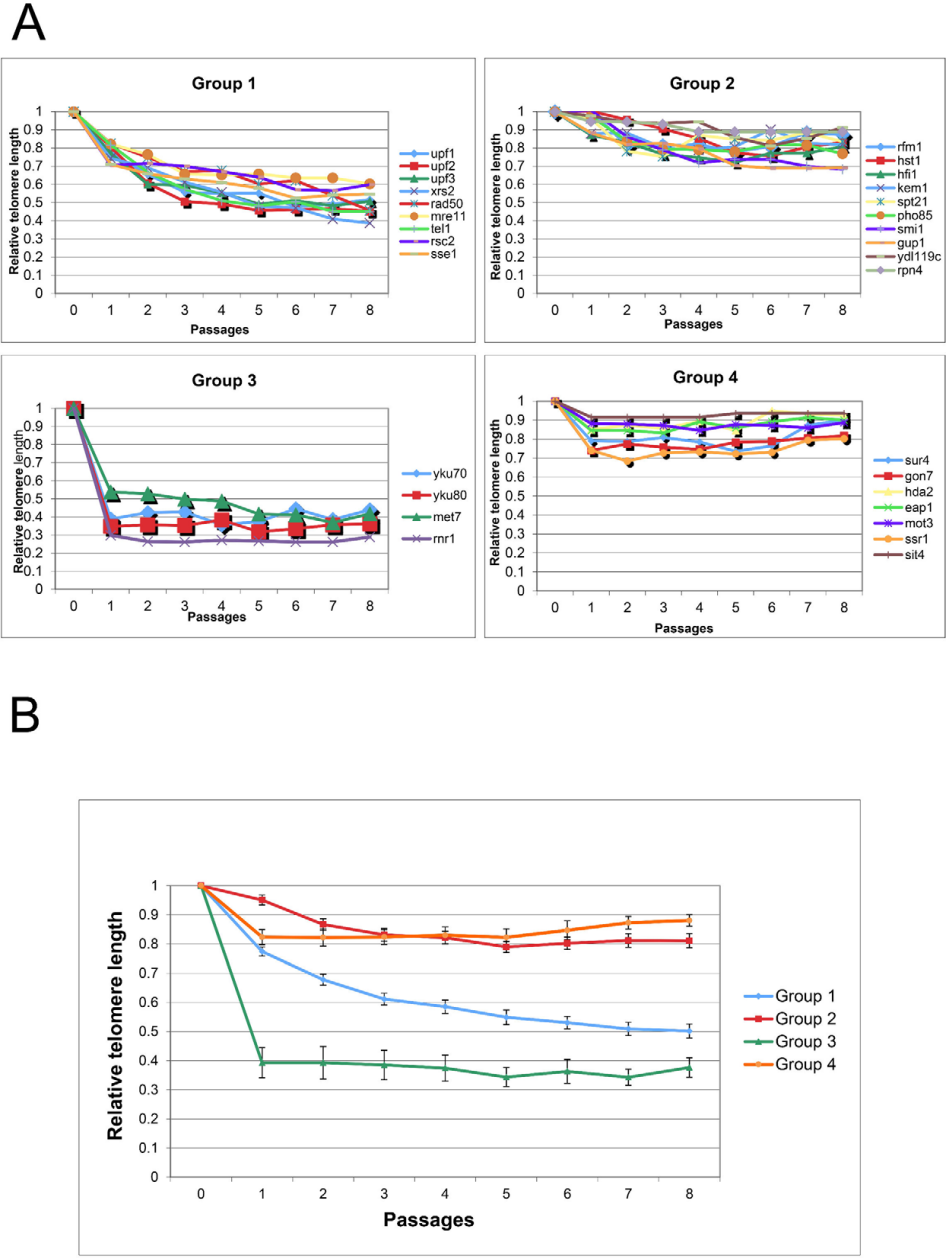
TELKA divides 30 *tlm* mutants into functional categories. (**A**) The *tlm* mutants can be clustered into four functional groups. A kinetic graph for each group is displayed. The X-axis represents the number of streaks (approximately 25 generations/streak) and the Y-axis represents telomere length relative to the wild-type length. (**B**) Mean of the kinetics of shortening of each group.

TELKA clustered all mutants into four groups, according to their kinetics of telomere shortening (Figure 2). The clusters obtained were compared to a million permutations of the same data, resulting in an empirical *P*-value <10^−6^. Reassuringly, mutants affecting different subunits of the same complex (e.g. Yku70 and Yku80) were clustered together (Figure 2).

The first group (Figure 2B) exhibits mild exponential shortening, which plateaus after approximately 5–6 streaks (125–150 generations). This mild exponential shortening kinetics (almost linear) suggests that the telomere length shortening is caused mainly by the end replication problem. One would therefore predict the components of this group to be directly involved in telomerase recruitment. Indeed, this first group includes mutants defective for the MRX complex (*mre11, rad50, xrs2*), as well as a deletion of the yeast ortholog of ATM, *TEL1* (27). Tel1 and the MRX complex function in a single pathway for telomere maintenance (28,29). Another member of the first group is *RSC2*, a component of the RSC chromatin remodeling complex (30). Rsc2 interacts physically and genetically with Tel1 and the MRX complex and is required for efficient recruitment of Tel1 and Mec1 to DNA break sites and for DNA end processing (31).

The second group (Figure 2B) exhibits linear shortening but arrives to its final length after approximately three streaks (75 generations). Moreover, the telomere end length in this group is on average 80% of the wild type. The linear shortening may imply an indirect role in telomerase recruitment or telomerase processivity. The second group includes mutants that affect chromatin configuration, such as members of the Sum1p/Rfm1p/Hst1p histone deacetylase complex required for origin-recognition-complex (ORC)-dependent silencing and mitotic repression and the Spt21 protein, part of the Spt10/Spt21 histone acetylase (32).

The third group (Figure 2B) includes only four components: the Ku heterodimer components (Yku70 and Yku80) (33), Met7 and Rnr1. Met7p is a folylpolyglutamate synthetase (FPGS) which works with the dihydrofolate synthetase (DHFS) Fol3p to add glutamyl side chains to *de novo* synthesized folate coenzymes (34). In the cytosol, folate coenzymes are involved in purine and thymidylate synthesis as well as in the biogenesis of the methyl group of methionine. In the mitochondria, 10-formyltetrahydrofolate is necessary for the formylation of the initiator tRNA and therefore for mitochondrial protein synthesis and maintenance of mitochondrial DNA (35). Rnr1 is the large subunit of a tetrameric RNR protein complex that catalyses the conversion of nucleotides to deoxynucleotides (36).

This group of mutants exhibited an acute telomere shortening visible after just one streak (25 generations). This extreme shortening kinetics may imply the involvement of nucleases in addition to failure in telomerase recruitment. Finding the Ku heterodimer in this group is not surprising due to its role in protection of the telomere against nucleolytic and recombinational activities (37). On the other hand, the presence of Met7 and Rnr1 in this group is intriguing.

The fourth group (Figure 2B) also shows rapid, yet mild, shortening; it includes the *gon7* mutant, defective for the KEOPS complex (38), as well as mutants lacking Sit4 and Eap1, two proteins implicated in the TOR signaling cascade (39). Although the second and fourth groups are similarly mild, they have distinctly different kinetics, with statistically significant separation (see Materials and Methods).

By iterating the clustering algorithm, it was possible to separate groups 1, 2 and 4 into coherent subgroups. In contrast, group 3 remained a single unit (Supplementary Figure S2).

### Met7 interacts genetically with the Ku heterodimer and has a physical interaction with Rnr1

We decided to further investigate group 3, composed of only four members:, *yku70Δ*, *yku80Δ*, *met7Δ* and *rnr1Δ* (Figure 2B). Whereas the Ku heterodimer is a well-established telomeric complex, the role of the other two genes in telomere length maintenance is unclear. First, we confirmed the identity of the mutants by PCR and complemented their telomere defects by transforming the mutants with plasmids carrying the wt genes (Supplementary Figure S3A). The *MET7* gene plays a role in the biosynthesis of methionine and also affects the biosynthesis of purines by cooperating with *FOL3* to add glutamic acid to folates (34). Mutations in additional methionine biosynthesis mutants (e.g. Met6, Met12, Met13) had no effect on telomere length, whereas a temperature-sensitive (*fol3–1*) mutation of the *FOL3* gene (encoding an essential enzyme, DHFS) led to shortened telomeres at the permissive temperature of 25°C (Supplementary Figure S3B). These results suggest that Met7 exerts its effect on telomere length maintenance by affecting nucleotide synthesis.

The RNR function is essential for life in all organisms (40). The *RNR1* gene has been found to be essential in several yeast backgrounds (e.g. W303). We found that BY4741 is able to survive in the absence of Rnr1 since it expresses a low but detectable level of *RNR3* expression. *RNR3* is a paralog of *RNR1* normally expressed only under DNA damaging conditions (36); in this strain background it appears to be leaky.

**Figure 3.**
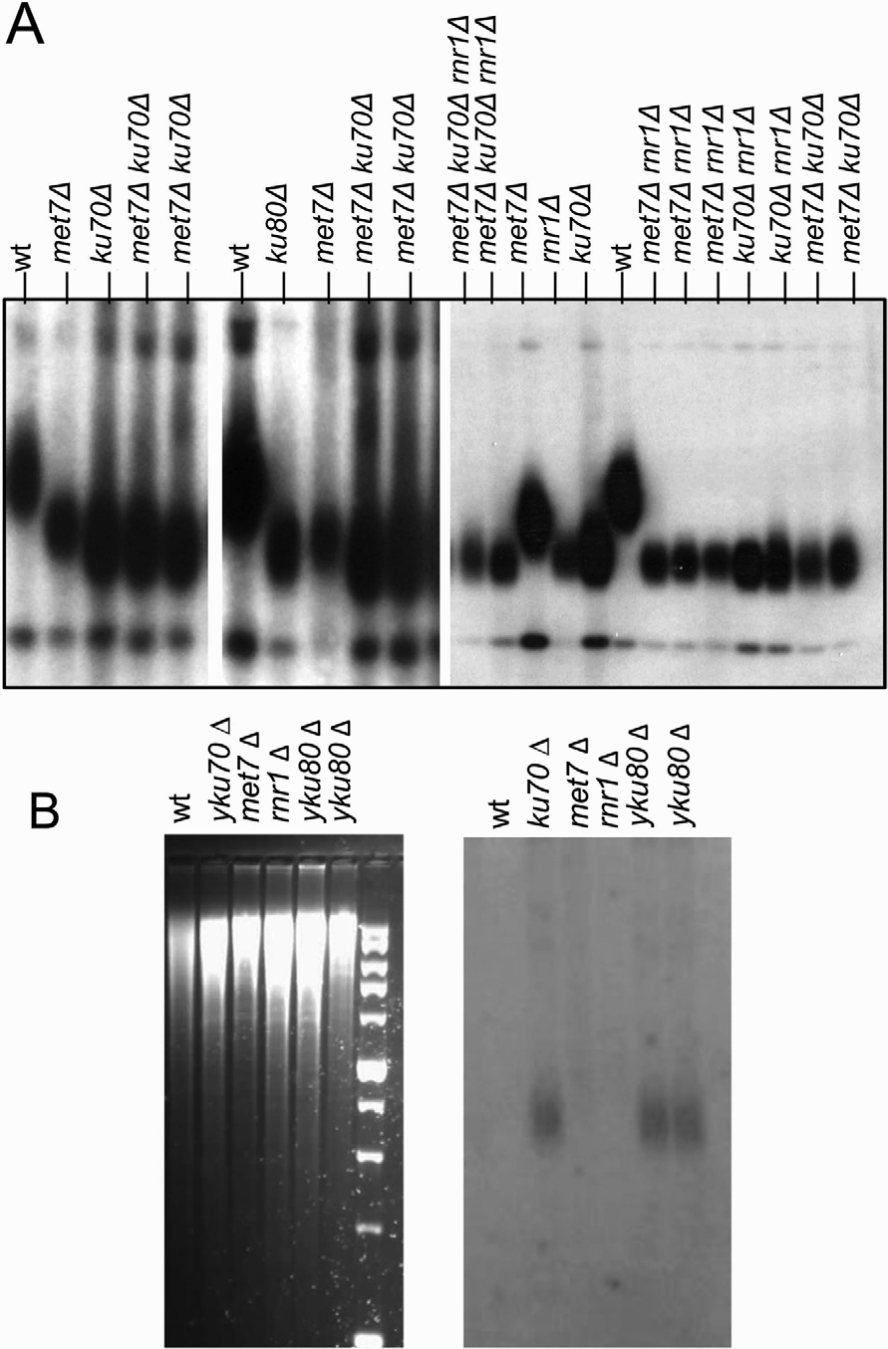
Telomeric phenotype of *met7Δ, rnr1Δ*, *yku70Δ* and *yku80Δ* mutants. (**A**) Teloblots of single, double and triple mutants. (**B**) Left panel: Ethidium bromide-stained gel. Right panel: In-gel Southern blot that hybridizes to the G-rich ssDNA telomeric overhang.

Figure 3A shows that telomeres are extremely short in *yku70Δ* and *rnr1Δ* mutants, and slightly longer in *met7Δ* strains, compared to the other mutants. To investigate the genetic relationships between the three genes, we created all double mutant combinations, as well as the triple *yku70Δ rnr1Δ met7Δ* mutant. As shown in Figure 3, *rnr1Δ* is epistatic to *yku70Δ*, *yku80Δ* and *met7Δ* for telomere length maintenance, confirming that the three mutants affect a single pathway/process. Mutants defective for the Ku heterodimer exhibit short telomeres, and an elongated ssDNA 3’ overhang (41) which can be detected by in-gel hybridization experiments, in which electrophoresis gels are hybridized, without denaturation, to a single strand probe (42). As Figure 3B shows, despite the similar short telomere lengths observed in the three mutants, only *yku70Δ* and *yku80Δ* exhibit long overhangs.

The Ku heterodimer plays central roles in telomere length maintenance, telomere structure and telomere position effect (43–45). One possible mechanism by which Met7 could be regulating telomeres is by affecting the cellular levels of the Ku components, or their recruitment to telomeres. We tagged Yku70 and Yku80 with a myc tag and performed chromatin immunoprecipitation essays in wt and *met7Δ* strains. Supplementary Figure S4A shows no significant differences between the *met7Δ* strain and the wild type. Similarly, western blot analysis shows no differences in the levels of Yku70 or Yku80 proteins (Supplementary Figure 4B). Similarly, the level of Rnr1 is not affected by mutations in *MET7* or *YKU70* (Supplementary Figure S5).

**Figure 4.**
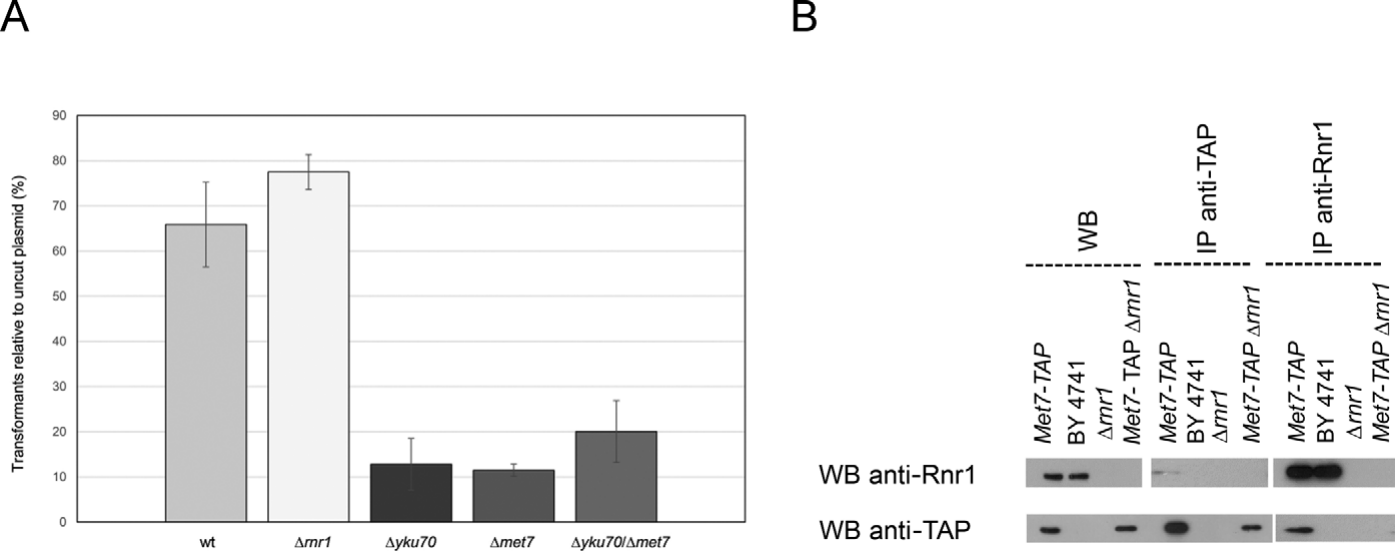
Role of *MET7* in NHEJ and co-IP of Met7 and Rnr1. (**A**) A plasmid digested with a linearizing restriction enzyme or an uncut plasmid were electroporated into different strains and their ability to repair by NHEJ was measured by counting colonies. The graph shows the ratio of transformants with the cut plasmid relative to the uncut plasmid. (**B**) Co-immunoprecipitation of Met7 and Rnr1. A strain carrying a TAP (Tandem Affinity Purification)-tagged Met7, an untagged wt strain (BY4741), a control strain deleted for *RNR1* and the same strain carrying a TAP-tagged Met7 were submitted to western blot analysis, before or after being immunoprecipitated with antibodies specific for the TAP tag, or anti-Rnr1. Rnr1 can be detected after anti-TAP immunoprecipitation, and Met7-TAP is detected upon immunoprecipitation of Rnr1.

### Met7 plays a role in NHEJ

In addition to their role in telomere biology, Yku70 and Yku80 have a crucial role in the repair of DSBs via NHEJ. When a DSB occurs, Yku70 and Yku80 are recruited and bind to the DNA ends to start the repair by NHEJ (46). When the *YKU70* or *YKU80* genes are deleted, the efficiency of NHEJ rate is drastically reduced (20). To test whether Met7 and Rnr1 may also play a role in NHEJ, we performed a ligation essay: a plasmid, either uncut or after digestion with a restriction enzyme that linearizes it, is transformed into yeast cells. NHEJ is required for *in vivo* ligation of the ends, resulting in a viable transformant. Thus, the ratio between the transformation efficiency of the cut and uncut plasmid represents a measurement of the ability of the cells to perform NHEJ. Figure 4 shows that *met7Δ* mutants, similarly to *yku70Δ* mutants, showed an extremely low NHEJ efficiency. Moreover, the *yku70Δ met7Δ* double mutant exhibited the same low efficiency, indicating that Met7 and the Ku heterodimer work together in NHEJ. In contrast, NHEJ is not affected by deletion of *RNR1* (Figure 4).

### Met7 physically interacts with Rnr1

Not much is known about the Met7 protein. In order to find physical interactors of Met7, we performed a genome-wide protein-fragment complementation assay (PCA). Briefly, a strain carrying a fusion between Met7 and a fragment of dihydrofolate reductase (DHFR) was mated to a library of fusions between each yeast open reading frame and a second DHFR fragment (47). If two proteins interact, the DHFR fragments are brought together in space and fold into the native structure, reconstituting the activity of the DHFR enzyme and allowing cells to proliferate in the presence of methotrexate (47). The PCA screen was carried out twice, once by mating the Met7-DHFR fusion to the MATa fusion collection and once by using the MATalpha fusion collection. A total of 12 proteins were found by this method to interact with Met7 (Table S2). Strikingly, Rnr1 was one of them. We confirmed the physical interaction between Rnr1 and Met7 by co-immunoprecipitation (Figure 4B). The physical interaction between Met7 and Rnr1 supports our TELKA results.

### Met7 and Yku70 affect dNTP levels

**Figure 5.**
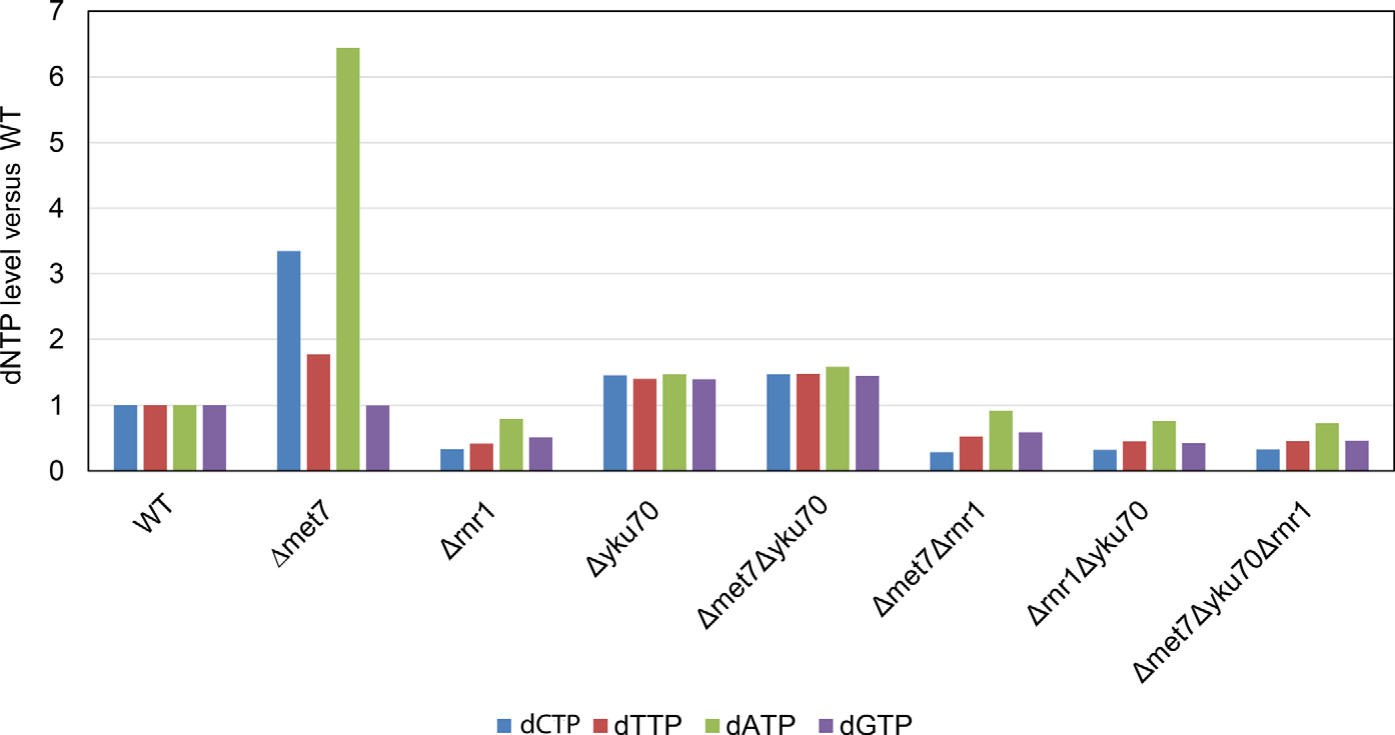
*RNR1, YKU70* and *MET7* affect dNTP synthesis. The relative levels of dNTPs are shown in single, double and triple mutants, compared to those of the wt.

In order to further investigate the connection between Rnr1, Met7 and Yku70, we measured the intracellular levels of dNTPs in the various single and double mutants, as well as in the triple *met7Δ yku70Δ rnr1Δ* mutant. In the absence of Rnr1, as expected, the level of dNTPs in the cell is low (ranging from 30% to 70% of the wt levels). The *met7Δ* strain, in contrast, exhibits a highly unbalanced dNTP profile with three- and six-times higher levels of dCTP and dATP than wt (Figure 5). It is interesting to note that the two dNTPs that are expressed at extremely high levels in the *met7Δ* mutants are dCTP and dATP, the two nucleotides not used by telomerase (which elongates the GT rich strand). This may suggest a mechanism by which excess of these two nucleotides may impair telomerase activity. *yku70Δ* mutants have very slightly elevated, almost normal levels of dNTPs. Surprisingly, deletion of *YKU70* suppresses the unregulated dNTP phenotype of *met7Δ.* This suppression depends on Rnr1 activity, as the *rnr1Δ met7Δ* and *rnr1Δ yku70Δ* double mutants, as well as the triple *met7Δ yku70Δ rnr1Δ* mutant, show a profile indistinguishable from that of the single *rnr1Δ* strain (Figure 5).

## DISCUSSION

Systematic genetic screens have uncovered a complex network of ∼400 genes that controls telomere length in yeast (11–13,48). The identification of the genes and the description of such a complex homeostasis constitutes the first step in our attempt to obtain a system-level portrayal of the mechanisms that maintain telomere length. Here we have used a new software, TelQuant, to accuretly measure telomere length, and we have developed a new method, TELKA, to cluster *tlm* mutants according to the dynamics of telomere length change.

We demonstrate the usefulness of TELKA by describing a new telomere pathway composed of the Rnr1 protein, with a role in dNTP synthesis, together with the Met7 FPGS and the Ku heterodimer. Using this novel method, we have linked genes not known before to work together in telomere biology and NHEJ; we have then confirmed genetic and physical interactions between the members of this telomere pathway.

The Ku heterodimer is pivotal for the repair of DSBs by NHEJ and also plays a variety of roles at telomeres, including telomere length regulation and 5′ end protection (49); these functions can be mutationally separated (41). Furthermore, some mutations that abolish an interaction with telomerase RNA do not affect 5′ end protection (50). The telomere shortening in *yku70Δ* or *yku80Δ* mutants is unlikely to be due to an indirect effect of Ku's role in NHEJ, as disruption of *LIG4*, the DNA ligase required for NHEJ, has no effect on telomere length (51). We have found that *MET7* collaborates with Ku and also plays important roles in both NHEJ and telomere length maintenance. We have ruled out a simple mechanism by which Met7 is required to maintain the levels of Yku70 or Yku80 proteins or their recruitment to telomeres (Supplementary Figure S2). Moreover, the long 3’ overhangs, which are the hallmark of *yku* mutants (49), are absent from the telomeres of *met7Δ* mutants (Figure 3B). It is still unclear why *yku70* and *yku80* mutants show extended overhangs; these are due, only in part, to resection by Exo1 (52). It is not clear why the absence of Ku allows for longer resection, or what prevents extensive, *cdc13–1*-like chromosomal resection. The lack of extended resection suggests that Met7 (and Rnr1) affects a stage prior to the one in which the Ku heterodimer prevents resection. The role played by Met7 in NHEJ seems to be unrelated to the dNTP levels of the cell: the double mutant *met7Δ yku70Δ* has almost normal levels of dNTPs, however it is as defective for NHEJ as the *yku70Δ* or the *met7Δ* single mutants. Met7 is able to modify proteins by adding glutamate residues; however, its range of substrates has not been mapped yet. The precise role of Met7 in the process will be the focus of future studies.

Folates are required for the biosynthesis of methionine, purines, dTMP and formyl-methionyl-tRNA, essential for the initiation of protein synthesis. In eukaryotic cells, folate coenzymes are compartmentalized primarily between the cytoplasm and the mitochondria and have parallel enzyme systems for the synthesis and interconversion of folate-bound one-carbon units, which are carried and donated by tetrahydrofolate derivatives. Intracellular folates are primarily found as poly-gamma-glutamates, which seem to be better substrates for folate-dependent enzymes and are also better retained in their compartments (34,35). Two enzymes are responsible for the binding of glutamate to folate derivatives. The DHFS (Fol3) adds the first glutamate to dihydropteroic acid, yielding dihydrofolate, whereas FPGS (Met7) adds sequentially other glutamates. Mutations in both enzymes affect telomere length; this mechanism also includes the large subunit of the ribonucleotide reductase, Rnr1. These results suggest a common mechanism involving dNTP formation. Indeed, our PCA and co-IP results indicate that a physical interaction exists between Met7 and Rnr1. Consistent with our results, a recent screen for mutants affecting the S-phase length found both *met7Δ* and *rnr1Δ* mutants (53), probably due to their imbalanced dNTP pool, which allows them to escape detection by the S-phase checkpoint (54).

Our results link the Yku complex and Met7 to dNTP metabolism: *rnr1Δ* mutants have a kinetic behavior similar to that of *met7Δ* and *yku* mutants, and they show epistasis for telomere length maintenance (Figures 2 and 3). The *rnr1Δ* mutants, as expected, have very low levels of dNTPs. We show that *met7Δ* mutants have unregulated levels of dNTPs that resemble some of the *rnr1* mutant alleles in which the allosteric regulation is affected (55). Surprisingly, deletion of *YKU70* suppressed the dNTP regulation defect of *met7Δ* mutants: the double mutants *met7Δ yku70Δ* showed normal dNTP levels, similar to those of the single *yku70Δ* strain (Figure 5). The phenotype of *met7Δ* and its suppression by deletion of *YKU70* were dependent on proper function of the *RNR1* gene, as the double mutants *rnr1Δ yku70Δ* and *rnr1Δ met7Δ* as well as the triple *rnr1Δ met7Δ yku70Δ* showed low dNTP levels (Figure 5). The short telomeres observed in these mutants, however, cannot be explained by a simple model in which lack of telomerase activity due to a low supply of dNTPs is responsible for telomere shortening. Despite their similar telomere length and shortening kinetics, the mutants have very different dNTP levels and ratios (Figure 5); thus, telomere length in this case does not seem to depend on a certain ratio between individual nucleotides (18). It has been recently shown that many DNA repair mutants exhibit long telomeres and high dNTP levels (56), as a consequence of induction of the DNA damage response. It is thus possible to interpret the high dNTP levels observed in *met7Δ* mutants as an indirect consequence of the checkpoint induction. Indeed, as stated above, both *rnr1Δ* and *met7Δ* were isolated in a systematic screen for mutants that exhibit elongated S-phase (53). According to this interpretation, the suppression of dNTP uneven levels by mutation of Yku components could be due to an effect in an intra-S checkpoint sub-pathway that affects dNTP production. These results are consistent with a previous report (57) that indicated that mutation of *YKU70* or *YKU80* can suppress the lethality of a strain deleted for the *MEC1* DNA damage response gene. The main role of *MEC1*, which encodes the yeast ATM orthologue, seems to be the regulation of dNTP levels during S-phase and following DNA damage (58,59).

We show complex genetic relations in the dNTP synthesis machinery: while *yku70Δ* suppresses the imbalanced dNTPs seen in *met7Δ*, *rnr1Δ* is epistatic to both *yku70Δ* and *met7Δ.* Our results therefore define *MET7* as an important gene with functions in dNTP biosynthesis, telomere maintenance and DNA repair. Changes in the dNTP pools are associated with spontaneous mutations and chromosome instability, as well as with resistance of tumor cells to drugs (60). Met7, like the Ku heterodimer and the RNR, is conserved in humans. Human FPGS, the ortholog of Met7, has been shown to play a central role in determining the success of anti-folate treatment against cancer cells (61). Further studies are required to test whether the pathway uncovered here is functionally conserved in humans.

## SUPPLEMENTARY DATA

Supplementary Data are available at NAR Online.

SUPPLEMENTARY DATA
