# Supplementary material for: Telomere length kinetics assay (TELKA) sorts the telomere length maintenance (tlm) mutants into functional groups
Source: Nucleic Acids Res. 2014 Apr 11;42(10):6314–25. doi: 10.1093/nar/gku267 (PMC4041441; doi:10.1093/nar/gku267)
Supplement: SUPPLEMENTARY DATA [file supp_42_10_6314__index.html]

Telomere length kinetics assay (TELKA) sorts the telomere length maintenance (tlm) mutants into functional groups — Telomere length kinetics assay (TELKA) sorts the telomere length maintenance (tlm) mutants into functional groups — SUPPLEMENTARY DATA 

# Telomere length kinetics assay (TELKA) sorts the telomere length maintenance (*tlm*) mutants into functional groups

## SUPPLEMENTARY DATA

**Files in this Data Supplement:**

- SUPPLEMENTARY DATA
