# Supplementary material for: Telomere length kinetics assay (TELKA) sorts the telomere length maintenance (tlm) mutants into functional groups
Source: Nucleic Acids Res. 2014 Apr 11;42(10):6314–25. doi: 10.1093/nar/gku267 (PMC4041441; doi:10.1093/nar/gku267)
Supplement: SUPPLEMENTARY DATA [file supp_gku267_nar-03658-f-2013-File007.pdf]

Suplementary Material

Content:

Supplementary Text1

Supplementary Table1

Supplementary Table2

Supplementary Tables and Figure Legends

Supplementary Text 1

TelQuant and Quantification of telomere length: The telomere band (Y' telomeres) signal (median and mean) is measured using the TelQuant software , by comparing its position on a teloblot to that of the control size bands (size 2044 and 779bp). TelQuant software was programmed using the VisualBasic6 programming environment.

TelQuant algorithm: Southern blots are hybridized with radioactive probes and exposed to a sensitive X-ray film. The areas of the X-ray film that were exposed to X-rays are seen as dark signals after the film is developed. Therefore, the Southern blot image is composed of dark areas of the detected signal and a relatively bright background. The TelQuant software scans the Southern blot's image's pixels, gets the RGB Decimal value of each scanned pixel and calculates the intensity for each pixel as 1/RGB Decimal value.

After loading a Southern blot image and manually fixing the 36 running lanes (a line running through the middle of the lane is defined as a Y-line, our blots usually contain 36 lanes, but this number can be adjusted), TelQuant scans each of the 36 Y–lines separately in a criss-cross manner. The scan distance (X) is calculated as (X=the distance between two Y-lines / 2). The scan is preformed horizontally from both sides of the Y-line starting from XY-line - X to XY-line + X. The horizontal scan is defined as X-line, and the vertical Y-line is scanned vertically X-line by X-line from the start of the Y-line to its end.

There are three layers of data processing. The first layer detects the peak signal/density value of the Y-line ('MaxSignal'). The second layer of processing compares each signal point (pixel) to the MaxSignal and grades it: if the point signal is greater than MaxSignal divided by the FilterValue, then it grades it as 1, otherwise, it grades it as 0. Therefore, the FilterValue, which is manually determined, enables TelQuant to process noisy images, requiring stringent filtering. In conclusion, the second processing layer creates a binary array (1,0) of the raw data (can be referred also as black/white filtering). The last processing layer sums up each X-line grade and searches for the longest consecutive segments with high grades. The longest segment is defined

as the telomere band. The other 2 segments are defined as the 2044 bp band (the upper size marker) and the 779 bp band (the lower size marker).

After the bands (segments) are located, TelQuant draws a histogram (‘Telogram’) of the analyzed raw data and finds the median of each band's histogram (MedianLine). The middle of each band (the mean of the band’s first detection point to the last detection point) is also calculated (MeanLine) and in most cases its location is very close to the MedianLine. The difference between the locations of both lines is defined as the AreaRatio:

AreaRatio=(MedianLine–first detection point)/(last detection point – first detection point) * 100.

AreaRatio different from 50% could be interpreted as a technical matter (unclean samples/images led to a biased detection), but in rare cases it represents a biological issue (e.g. most signal is located preferably in the lower region of the telomere smear, implying that there is a preference for shorter or longer telomeres in the strain examined).

In an optimal Southern blot image, all of the marker bands should be found automatically. Unfortunately, not all the images are optimal, and bands with weak signals or a strong background can be missed or wrongly localized (especially when the MarkerBands are overrun by the telomere smear). The MarkerBands algorithm therefore suggests the missed bands’ location as the median location of the nearest detected neighbor bands. As a last resort, TelQuant offers the possibility of manually fixing the bands. The possibility of allocating individual MarkerBands for each line enables TelQuant to deal with distorted lanes of all sorts.

After all bands in the teloblot are identified, TelQuant translates the TelomereBands into molecular weight (base pairs). During electrophoresis through an agarose gel, linear DNA fragments in the Ogston-sieving domain migrate at a rate that is inversely proportional to the logarithm of their molecular weight. TelQuant uses this observation and constructs a graph that relates the migration distance (Y-pixels locations) to the base-pair size of the fragment using the migration distance of the 779 and the 2044 bands. After the migration to base pairs graph is constructed, TelQuant calculates the TelomereBands size (in base pairs) from the graph. Finally, all calculations are exported into an embedded Excel sheet (dynamic link between the content of TelQuant software and the content in a Microsoft Office Excel workbook) for additional

processing. When digesting the yeast DNA with XhoI, the terminal telomeric fragment carries 900 bp of sub-telomeric information, that is substracted prior to further mathematical processing of the TelomereBand values.

Supplementary Table 1: Strains analyzed by TELKA

Δupf1	*MAT*a *ura3Δ met15Δ leu2Δ his3Δupf1*::KanMX
Δupf2	*MAT*a *ura3Δ met15Δ leu2Δ his3Δupf2*::KanMX
Δupf3	*MAT*a *ura3Δ met15Δ leu2Δ his3Δupf3*::KanMX
Δxrs2	*MAT*a *ura3Δ met15Δ leu2Δ his3Δxrs2*::KanMX
Δrad50	*MAT*a *ura3Δ met15Δ leu2Δ his3Δrad50*::KanMX
Δmre11	*MAT*a *ura3Δ met15Δ leu2Δ his3Δmre11*::KanMX
Δtel1	*MAT*a *ura3Δ met15Δ leu2Δ his3Δtel1*::KanMX
Δrsc2	*MAT*a *ura3Δ met15Δ leu2Δ his3Δrsc2*::KanMX
Δsse1	*MAT*a *ura3Δ met15Δ leu2Δ his3Δsse1*::KanMX
Δrfm1	*MAT*a *ura3Δ met15Δ leu2Δ his3Δrfm1*::KanMX
Δsum1	*MAT*a *ura3Δ met15Δ leu2Δ his3Δsum1*::KanMX
Δhst1	*MAT*a *ura3Δ met15Δ leu2Δ his3Δhst1*::KanMX
Δhfi1	*MAT*a *ura3Δ met15Δ leu2Δ his3Δhfi1*::KanMX
Δkem1	*MAT*a *ura3Δ met15Δ leu2Δ his3Δkem1*::KanMX
Δspt21	*MAT*a *ura3Δ met15Δ leu2Δ his3Δspt21*::KanMX
Δpho85	*MAT*a *ura3Δ met15Δ leu2Δ his3Δpho85*::KanMX
Δsmi1	*MAT*a *ura3Δ met15Δ leu2Δ his3Δsmi1*::KanMX
Δgup1	*MAT*a *ura3Δ met15Δ leu2Δ his3Δgup1*::KanMX
Δydl119c	*MAT*a *ura3Δ met15Δ leu2Δ his3Δydl111c*::KanMX
Δrpn4	*MAT*a *ura3Δ met15Δ leu2Δ his3Δrpn4*::KanMX
Δsur4	*MAT*a *ura3Δ met15Δ leu2Δ his3Δsur4*::KanMX
Δgon7	*MAT*a *ura3Δ met15Δ leu2Δ his3Δgon7*::KanMX
Δhda2	*MAT*a *ura3Δ met15Δ leu2Δ his3Δhda2*::KanMX
Δeap1	*MAT*a *ura3Δ met15Δ leu2Δ his3Δeap1*::KanMX
Δmot3	*MAT*a *ura3Δ met15Δ leu2Δ his3Δmot3*::KanMX
Δssr1	*MAT*a *ura3Δ met15Δ leu2Δ his3Δssr1*::KanMX
Δsit4	*MAT*a *ura3Δ met15Δ leu2Δ his3Δsit4:*:KanMX
Δku70	*MAT*a *ura3Δ met15Δ leu2Δ his3Δyku70*::KanMX
Δku80	*MAT*a *ura3Δ met15Δ leu2Δ his3Δyku80*::KanMX

Δmet7	*MAT*a *ura3Δ met15Δ leu2Δ his3Δmet7*::KanMX
Δmot2	*MAT*a *ura3Δ met15Δ leu2Δ his3Δmot2*::KanMX
Δgpb2	*MAT*a *ura3Δ met15Δ leu2Δ his3Δgpb2*::KanMX
Δpsy4	*MAT*a *ura3Δ met15Δ leu2Δ his3Δpsy4*::KanMX

Supplementary Table 2

Physical interactors of Met7

Met6	Cobalamin-independent methionine synthase, involved in methionine biosynthesis and regeneration.
Ino1	Inositol-3-phosphate synthase, involved in synthesis of inositol phosphates and inositol-containing phospholipids
Eno1	Enolase I, catalyzes the conversion of 2-phosphoglycerate to phosphoenolpyruvate during glycolysis and the reverse reaction during gluconeogenesis.
Fba1	Required for glycolysis and gluconeogenesis; locates to mitochondrial outer surface upon oxidative stress.
Cdc19	Pyruvate kinase, functions as a homotetramer in glycolysis to convert phosphoenolpyruvate to pyruvate.
Mrf1	Mitochondrial translation release factor, involved in stop codon recognition and hydrolysis of the peptidyl-tRNA bond during mitochondrial translation.
Thr1	Homoserine kinase, conserved protein required for threonine biosynthesis. Expression is regulated by the Gcn4 general amino acid pathway.
Pef1	Penta-EF-hand protein required for polar bud growth and cell wall.
Asc1	G-protein beta subunit inhibitor of Gpa2p; ortholog of RACK1 that inhibits translation; core component of the small (40S) ribosomal subunit.
Rpl7B	Protein component of the large (60S) ribosomal subunit, contains a conserved C-terminal Nucleic acid Binding Domain (NDB2)
Dug1	Cys-Gly metallo-di-peptidase; forms a complex with Dug2p and Dug3p to degrade glutathione (GSH).
Sec63	Hsp40/DnaJ family that regulates Hsp70 chaperone activity, protein targeting and import into the ER.
Rnr1	Major isoform of the large subunit of the ribonucleotide-diphosphate reductase; the RNR complex catalyzes rate-limiting step in dNTP synthesis.

Supplementary Legends:

Supplementary Table1: List of *TLM* mutant strains used in our kinetics assay.

Supplementary Table2: Physical interactors of Met7 uncovered by Genome-wide protein-fragment complementation assay (PCA). Green labeled - metabolic proteins. Pink labeled – ribosomal proteins.

Supplementary Figure 1: Clustering of known complexes according to telomere kinetics. **A.** Clustering results of known complex components. Sixteen *tlm* mutants known to affect well-defined protein complexes were re-created after meiosis and subjected to 9 consecutive passages (~225 generations). Colonies were re-streaked only upon reaching a standard size to ensure equal number of cell divisions. TELKA was used to cluster them into groups; the results fit the known correspondence to physical complexes. The branch containing long *tlm* mutants is marked in red. **B., C.** Genes belonging to the same complex share similar telomeric kinetics. **B.** Three mutants affecting to the NMD complex exhibit similar rates of telomere shortening. **C.** Three mutants inactivating the N-acetyltransferase NatC show similar kinetics of telomere elongation.

Supplementary Figure 2: Subgrouping of *tlm* mutants. Mutants in each of the groups were clustered according to similarity in kinetics. Only group 3 could not be sub-divided further.

Supplementary Figure 3: A. Complementation of the telomeric phenotype of *met7Δ* by a plasmid carrying the wt *MET7* gene. **B.** A *fol3-1* temperature-sensitive mutant exhibits short telomeres (similar in length to those of *met7Δ* strains) at the permissive temperature (25°C).

Supplementary Figure 4: Yku levels and localization is not affected in *met7Δ* mutants. A. RT-PCR-telomeric ChIP assay comparing binding of myc-tagged Yku70 and YKu80 to telomeres in *met7Δ* mutant vs wild type. The non-telomeric *ARO1* locus was used to normalize the relative levels. The relative fold enrichment\depletion of the telomere-associated proteins Yku70 and Yku80 was calculated as follows: [tel-IP/*ARO1*-IP]/[tel-input/*ARO1*-input]. **B.**

Western blot with an anti-GST antibody. Protein levels of GST-tagged Yku70 or Yku80 in wt or *met7Δ* strains were measured.

Supplementary Figure 5: Rnr1 protein levels are not altered in the absence of Met7 or Ku70. Western blot with anti-Rnr1 antibody. Protein levels of Rnr1 were measured in *met7*Δ and *yku70*Δ mutants.

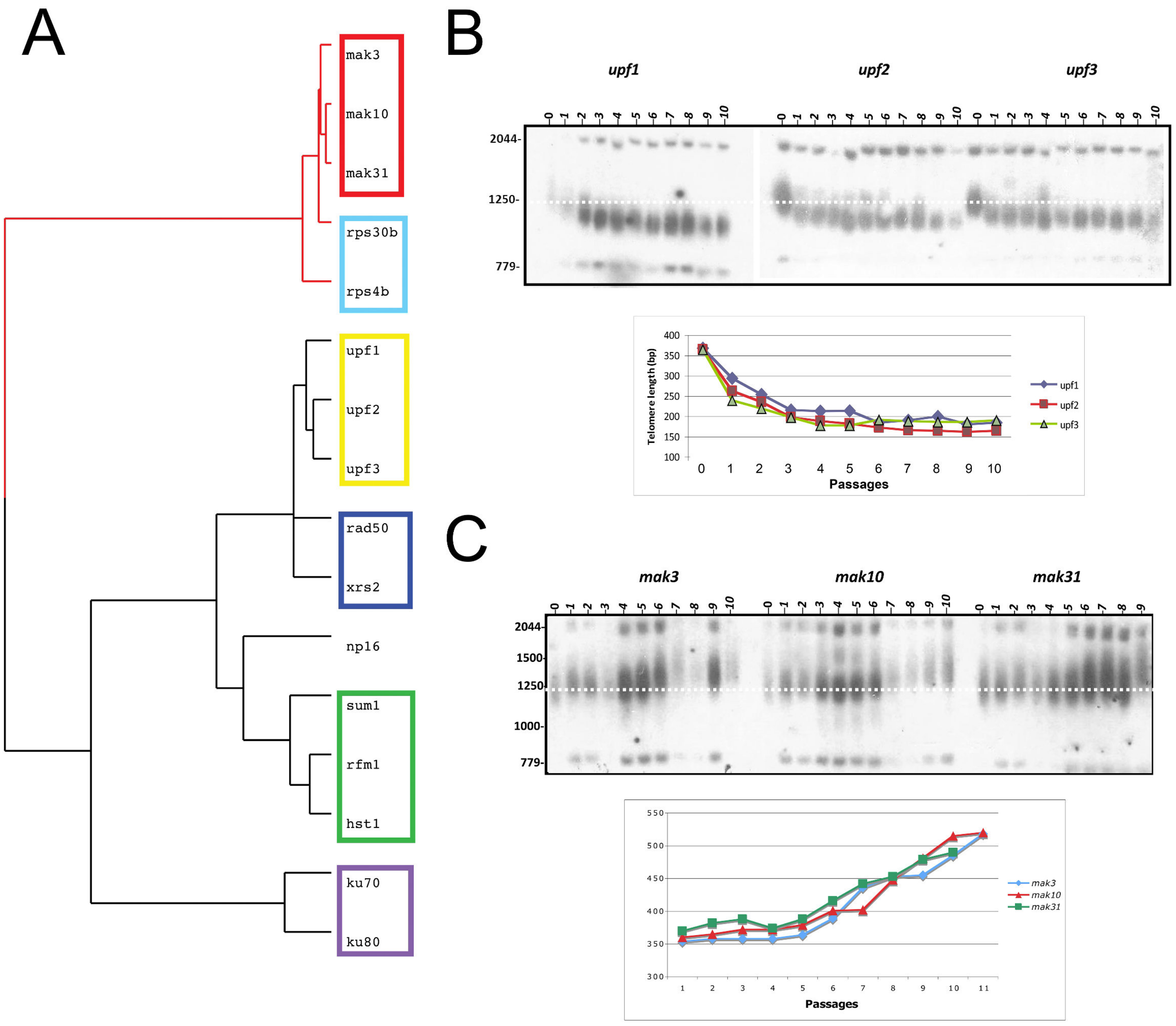

Rubinstein et al., Suppl. Fig. 1

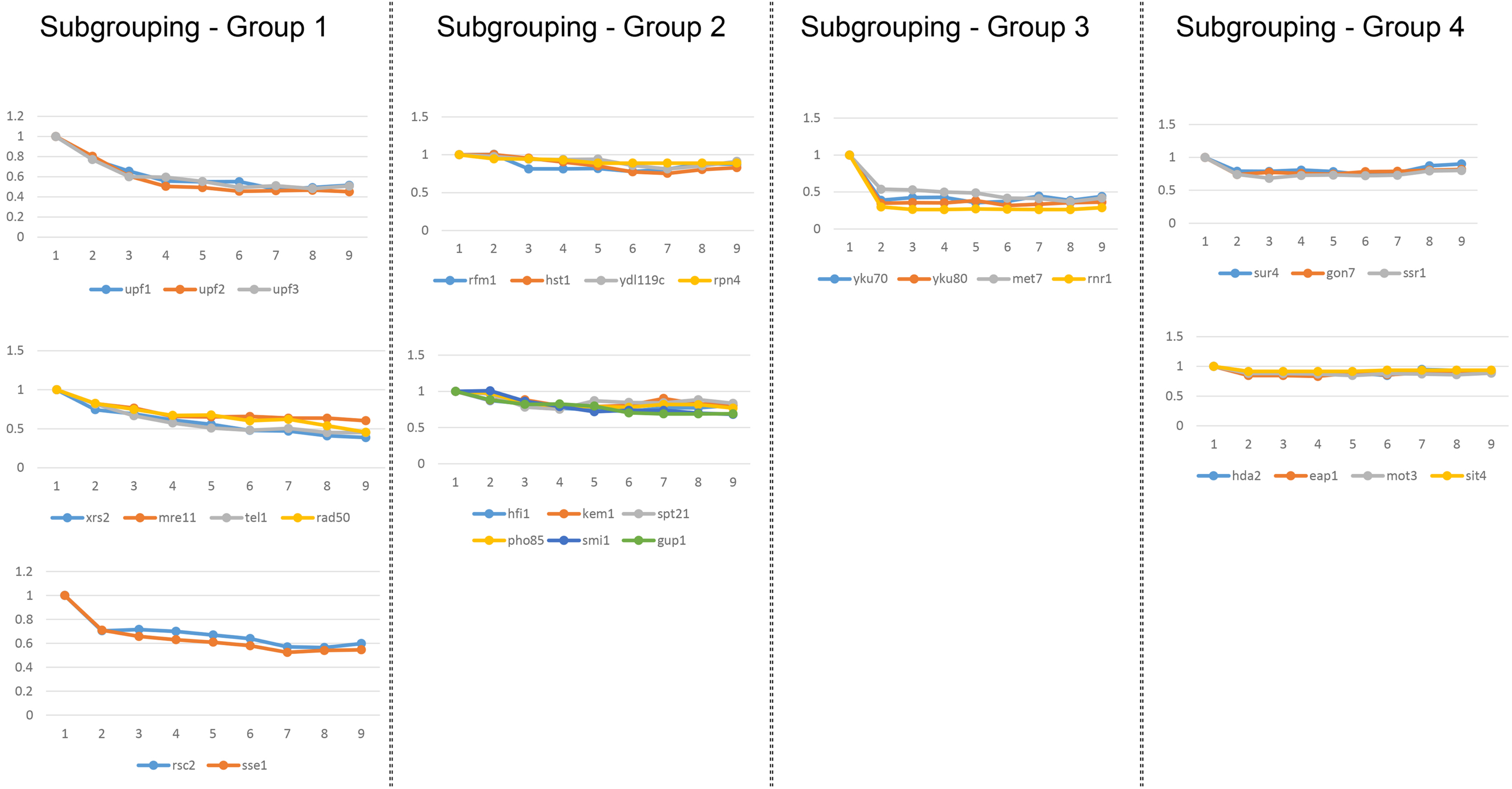

Rubinstein *et al.*, Supplementary Figure 2

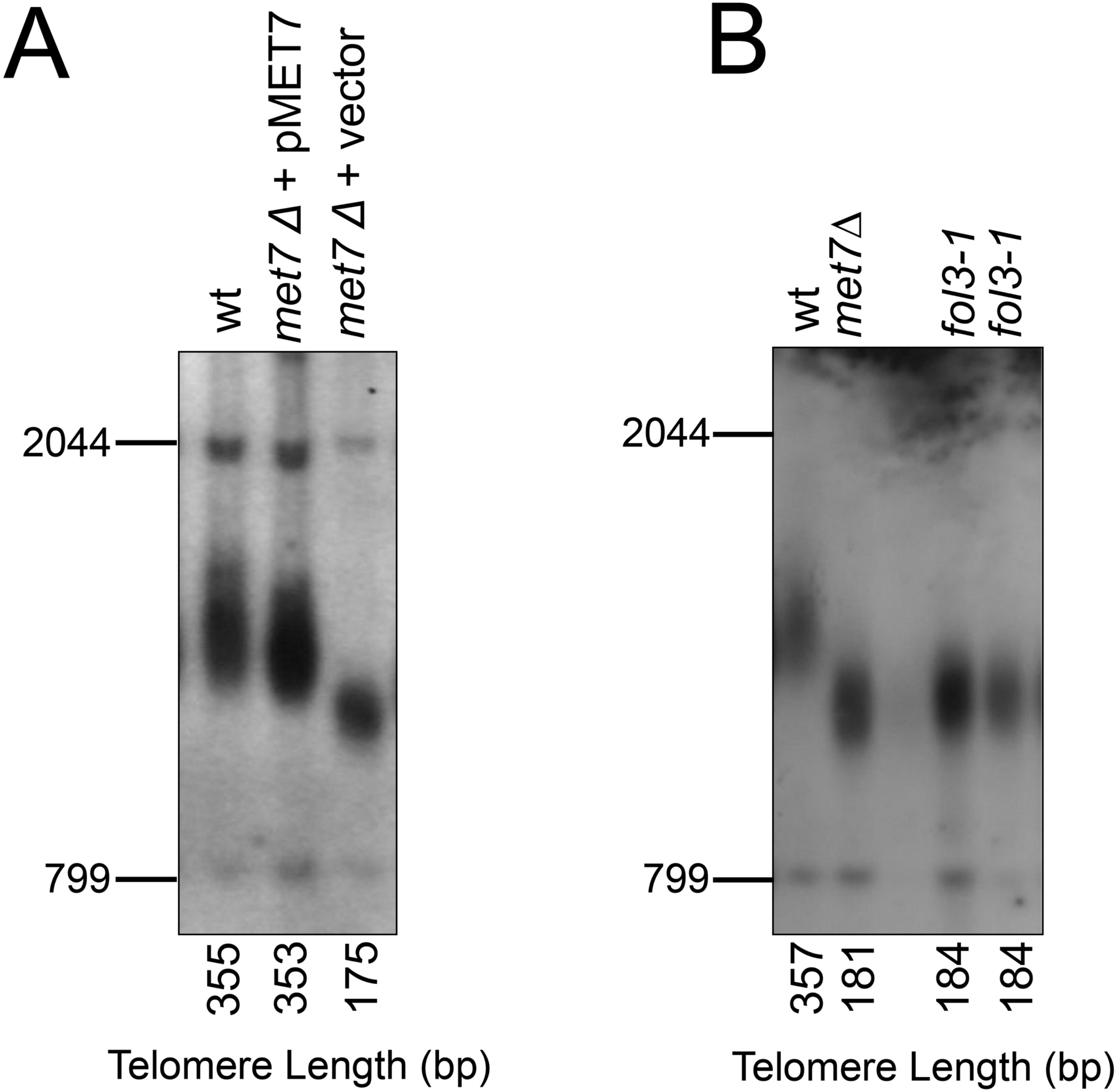

Rubinstein et al., Suppl. Fig.3

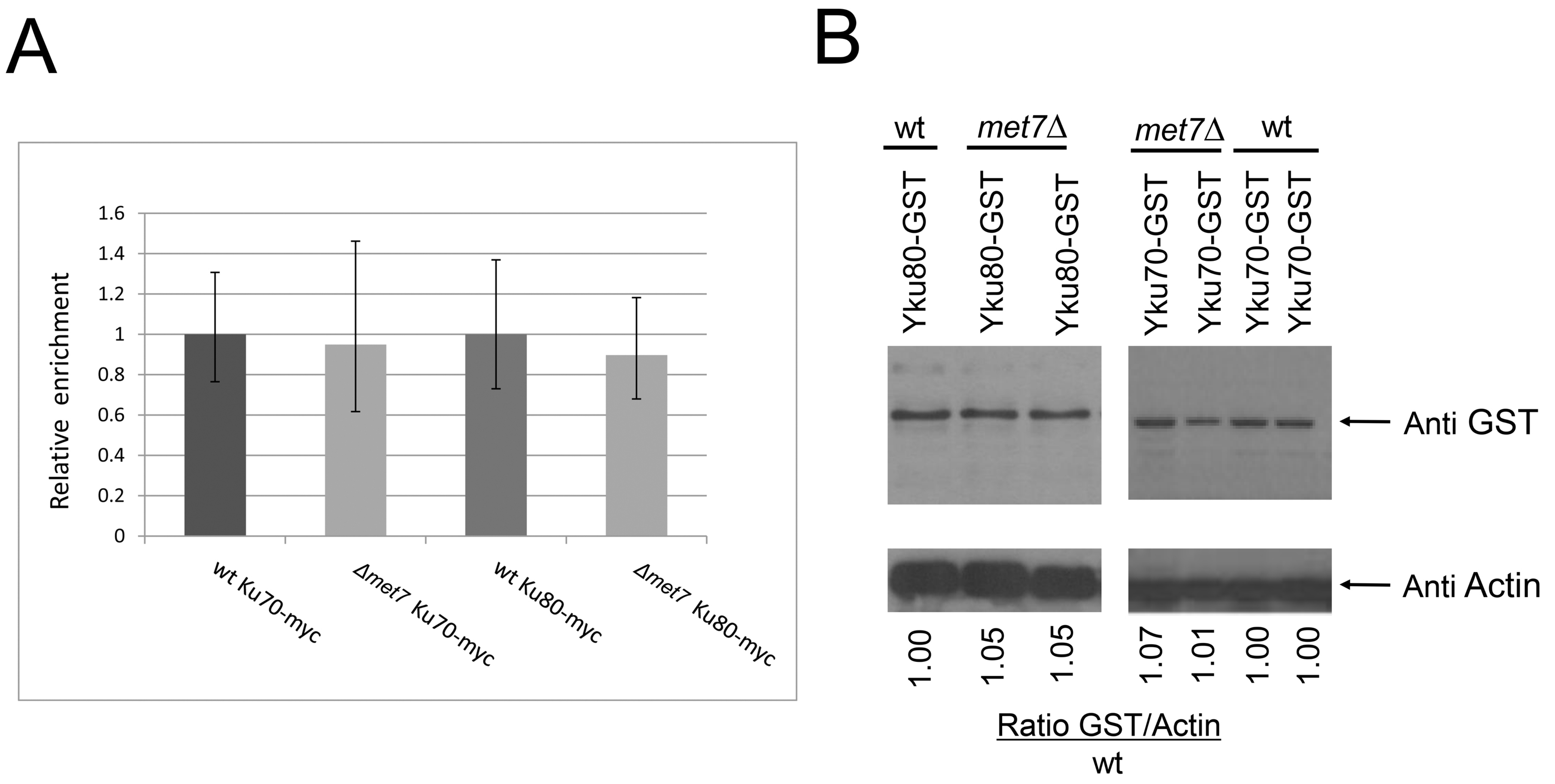

Rubinstein et al., Suppl. Fig.4

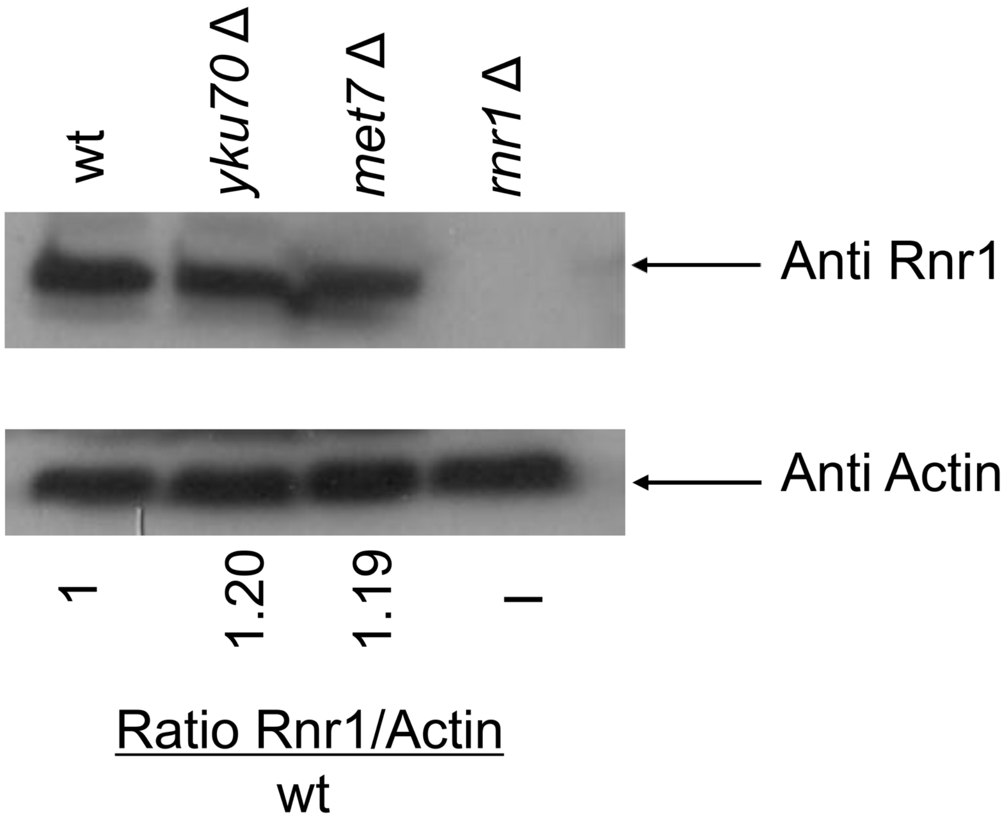

Rubinstein et al., Suppl. Fig.5
